# (Bio)degradable Polymeric Materials for Sustainable Future—Part 2: Degradation Studies of P(3HB-*co*-4HB)/Cork Composites in Different Environments

**DOI:** 10.3390/polym11030547

**Published:** 2019-03-22

**Authors:** Sebastian Jurczyk, Marta Musioł, Michał Sobota, Magdalena Klim, Anna Hercog, Piotr Kurcok, Henryk Janeczek, Joanna Rydz

**Affiliations:** 1 Institute for Engineering of Polymer Materials and Dyes, Paint and Plastics Department in Gliwice, 50A Chorzowska St., 44-100 Gliwice, Poland; sebastianjurczyk@vp.pl; 2 Centre of Polymer and Carbon Materials, Polish Academy of Sciences, 34. M. Curie-Skłodowska St., 41-819 Zabrze, Poland; m.sobota@cmpw-pan.edu.pl (M.S.); klim.magdalena@gmail.com (M.K.); ahercog@cmpw-pan.edu.pl (A.H.); p.kurcok@cmpw-pan.edu.pl (P.K.); h.janeczek@cmpw-pan.edu.pl (H.J.); jrydz@cmpw-pan.edu.pl (J.R.); 3 Department of Microbiology and Virology School of Pharmacy with the Division of Laboratory Medicine Medical University of Silesia, 4 Jagiellońska St., 41-200 Sosnowiec, Poland

**Keywords:** poly(3-hydroxybutyrate-*co*-4-hydroxybutyrate), composites, cork, (bio)degradation, composting

## Abstract

The degree of degradation of pure poly(3-hydroxybutyrate-*co*-4-hydroxybutyrate) [P(3HB-*co*-4HB)] and its composites with cork incubated under industrial and laboratory composting conditions was investigated. The materials were parallelly incubated in distilled water at 70 °C as a reference experiment (abiotic condition). It was demonstrated that addition of the cork into polyester strongly affects the matrix crystallinity. It influences the composite degradation independently on the degradation environment. Moreover, the addition of the cork increases the thermal stability of the obtained composites; this was related to a smaller reduction in molar mass during processing. This phenomenon also had an influence on the composite degradation process. The obtained results suggest that the addition of cork as a natural filler in various mass ratios to the composites enables products with different life expectancies to be obtained.

## 1. Introduction

In recent years, many efforts have been made to design composites with the advantages of being stable during use and being susceptible to microbial attack during organic recycling [1]. The addition of natural fillers to composites has been widely discussed in the literature because of their effect on the mechanical properties of composites. Moreover, it has been proven that natural fillers influence the degradation ability of these composites [2].

A material prepared by combining two or more constituents is called a composite material. Such multicomponent materials are gaining more interest for applications because of many advantages, such as low mass, high strength, and most importantly, low maintenance costs, of industrial appliances [3]. It is worth mentioning that even concrete and steel are currently being replaced by composite materials. In addition to the building industry, where composites are applied in both structural (e.g., bridges and roof structures) and nonstructural constructions (e.g., windows and door frames), they are also used in structural parts of a vehicle’s body (e.g., dashboard fascia and scooter frames) in the automotive industry [2].

There is, however, a critical limitation in terms of the reuse and recycling of composites prepared with a conventional polymer as a matrix [4]. However, natural composites may cause serious problems in the area of construction materials because of their easily degradable nature, although this characteristic is crucial for short-life applications. Environmental protection is also afforded as an advantage of composites made of biodegradable polymers as the matrix and reinforcement made of natural fillers [5].

Biodegradable plastics undergo relatively rapid degradation, and this process takes several months to several years to complete (depending on the material and degradation conditions). These plastics are biodegradable according to the European Union Standards (EN 13432) [6], and they can be organically recycled in compost. Biodegradable composites made of polylactide (PLA) are commonly used in food packaging; however, a balance between the mechanical and physical properties of these composites and their rapid degradation during composting is still required. Moreover, the food packaging industry demands intensive exploration and study regarding the use of biodegradable plastics and composites. Polyhydroxyalkanoates (PHAs) have a very important advantage for composite applications, namely because their polar character causes better adhesion to lignocellulose fiber [7]. PHA composites with vegetable or grain fillers may find an application in horticulture as environmentally friendly low-cost plastic crop containers intended to replace conventional ones. The main advantage of this solution is the biodegradation in soil after application [8]. In food packaging applications, where the container should be degraded at the same time as the food product, such composites can be used for the packaging of fresh fruits or vegetables [9]. Packages made of biodegradable plastics can be composted in household composters. According to the respective EU directives, the amount of biodegradable municipal waste stored in landfills must be reduced. It is well known that compostable plastics provide environmental sustainability, because they are produced from renewable raw materials. This contributes to reductions in the use of nonrenewable petrochemical materials [10].

Many research groups have confirmed the utility of biodegradable polymers as matrices for composites with natural fillers [11,12]. The addition of natural fillers to composite materials tends to improve the materials’ properties and lower the price [13]. Polyester mixed with cellulose-based fillers afford an economical material. Flax, sisal, jute, and wooden powder are considered as environmentally friendly fillers in engineering composites [14]. Gatenholm et al. showed that cellulose fibers have no significant influence on the thermal properties and crystallinity of PHB. Moreover, they also found that PHB composites with wheat straw, depending on the filler content, present much better or similar mechanical properties compared with neat matrix. [15] In the poly(3-hydroxybutyrate-co-3-hydroxyvalerate) (PHBV) composites, increased contents of 3-hydroxyvalerate units as well as fiber contents in the matrix influences their mechanical strength, elasticity, and crystallization kinetics [16]. The use of flax, cellulose, abaca, and jute fibers in PHBV composites as a filler resulted in an increase in the tensile strength and tensile stiffness modulus at the observed drop in elongation at the break [11,17,18]. Luo and Netravali described the mechanical and thermal properties of poly(3-hydroxybutyrate-*co*-4-hydroxybutyrate) [P(3HB-*co*-4HB)] composites with fibers obtained from pineapple leaves depending on the fiber direction [19]. Composites with fibers arranged in the longitudinal direction were characterized by higher values of tensile strength and Young’s modulus as compared to the unfilled matrix, while for the transverse direction of the fibers, a decrease in the obtained values was observed. The values of the bending strength and flexural modulus increased with the increase of the longitudinal direction of fibers. Semicrystalline PHAs exhibit comparable properties with polypropylene and can replace it in some applications, especially in the packaging and automotive industries.

Cork is a versatile material of natural origins. It is a material containing extractives, suberin, lignin, waxes, tannins, and polysaccharides (cellulose and hemicellulose), but cellulose does not play a main role in cork’s mechanical properties as in other wood fillers [20]. This natural filler has excellent properties, such as a low mass, density, and thermal coefficient; good electrical, thermal, acoustic, and vibration insulation; buoyant ability; and impermeability to gases and liquids. It also exhibits elasticity and deformation without fracturing under compression and has considerable durability. These advantages of cork along with its renewability, possibility of incinerating with energy recovery, and reduced concerns about health and safety make it an interesting alternative as a filler in biodegradable composites [20,21,22]. Composite materials also provide low abrasive wear to processing equipment, such as extruders and molds. Additionally, high adhesion between fillers and the matrix of composites is important as it influences the properties of the final product. Although fillers tend to be stable during the degradation process in soil, they probably allow microorganisms to attack the composite [23].

The degree of degradation of a composite is relevant to determine the shelf life of packaging applications. The presence of natural fillers in the obtained composites affects the degradation degree; this feature enables the time for which the packaging can be used to be determined. In the last few years, the range of biodegradable plastics has expanded incredibly in terms of both commercially available and under development products. Furthermore, technological progress has enabled the production of bio-based polymers, meeting the demand for environmentally friendly products. These features of biodegradable polymers have led to the development of strategies for replacing petrochemical feedstock with bio-based ones. The main goal of such development was to provide the market with biodegradable polymers with a variety of properties that are suitable for specific applications. Currently, the application of bio-based polymers in the packaging sector is restricted to food products and organic waste. An interesting alternative for composite application is the possibility of packaging charge-sensitive electronic circuit boards by using PHA/graphene composites. Composites with graphene used as a filler show considerable improvements in material stiffness and electrical conductivity, and these composites may be used in charge dissipating floor coverings for use in laboratories with AC/DC standards, where the equipment is very sensitive to electrostatic discharge [24,25]. The broadened horizons in ecological and economical areas should lead to an increase in the market of bio-based materials. Biodegradable materials can undergo controlled biological decomposition. This property has led to the development of safe and environmentally friendly products that are not harmful to human life and health, as opposed to traditional polymer waste stored in landfills that threaten the surrounding environment. The introduction of biodegradable composites in packaging into the market requires many scientific uncertainties to be overcome and many experimental studies to fully understand how such materials can be applied in daily life [26].

This paper presents the results of comparative studies on the biodegradation of pure P(3HB-*co*-4HB) containing 8 mol% of 4HB units and its composites with cork under industrial and laboratory composting conditions. P(3HB-*co*-4HB) was chosen as a polymer matrix in the presented studies due to its good mechanical properties, high elasticity, low crystallinity, and low melting temperature, compared with the PHB, the most common type from the PHAs family [27]. The materials were degraded under real industrial composting conditions in the sorting and composting plant in Zabrze. To verify the composting conditions and their influence on the degradation rate of the material, two industrial composting systems were applied: BIODEGMA and KNEER Collateral, abiotic degradation tests in distilled water were carried out under laboratory conditions. The addition of cork to the P(3HB-*co*-4HB) matrix had a marked influence on the degradation process. The changes in samples during the degradation process were monitored by visual observation and changes in the molar mass and thermal property (*T_max_*, *T*_5%_, *T_g_*, *T_m_*, *∆H_m_*).

## 2. Materials and Methods

### 2.1. Materials

Cork powder with an average particle size of 1.0 mm, bulk density of 65 kg·m^−3^, and humidity value of approximately 4.6% was provided by Corkpol, Ożarów Mazowiecki, Poland. P(3HB-*co*-4HB) in powdered form, with a number-average molar mass of *M_w_* = 625,000 g/mol and a molar mass dispersity of *M_w_*/*M_n_* = 2.5 estimated by gel permeation chromatography (GPC) (with PS standards) with 8% of 4-HB units (calculated by ^1^H NMR) under the trade name, Sogreen 00A, was obtained from Tianjin GuoYun Biological Material Co. Ltd., Tianjin, China.

### 2.2. Cork Preparation

Cork powder with an average particle size of 0.35 mm was obtained using the cryogenic grinder, SPEX SamplePrep 6870 model, equipped with a metal crucible, with a precooling time of 2 min and core crushing rate of 15 beats/min. The size of the prepared cork powder was measured using the laser analyzer, Coulter LS230, equipped with a dry measurement module to analyze the grain size in order to control the degree of specimen fragmentation. Before compounding, the cork powder was dried at 80 °C in a vacuum oven (Memmert, Schwabach, Germany).

### 2.3. Compounding and Processing

The composites based on cork powder as a filler and pure P(3HB-*co*-4HB) matrix were prepared using the micro-extruder, MiniLab (Thermo-Haake, Austin, TX, USA), equipped with corotating twin screws. The rate of screw rotation was 100 rpm. The type 1BA test specimen according to the ISO 527–2 standard [28] was prepared in the MiniJet (Thermo-Haake, Austin, TX, USA) mini injection molding machine. The mold temperature was set at 60 °C. The specimen processing parameters are presented in Table 1.

### 2.4. Degradation Environments

#### 2.4.1. Degradation under Industrial Composting Conditions

The study of organic recycling was carried out at the sorting and composting plant in Zabrze. The research was conducted in two industrial composting systems: KNEER and BIODEGMA. KNEER (container) is an intensive composting system with closed containers that are connected to a biomass aeration system. The container system contains leaves, branches, grass, and collected domestic organic waste from selected segregation. During the process, water circulates in a closed cycle. The generated gases are released to the atmosphere through a biological filter. The BIODEGMA composting system provides simple, economic, and efficient technological solutions to biodegrade organic waste, including separately collected biogenic and garden waste, sewage sludge, and the organic component of mixed municipal waste.

#### 2.4.2. Degradation under Laboratory Composting Conditions

The biodegradation process under laboratory aerobic conditions was performed using the Micro-Oxymax respirometer (Columbus Instruments S/N 110315) in mature compost prepared in accordance with the guidelines of composting technology used at the sorting and composting plant in Zabrze. For the respirometric test, samples with an average mass of 2.4 g were placed in glass jars containing 300 g of mature compost at a humidity of 40% and pH of 7.9 and then incubated at a temperature of 58 °C (±2 °C) for 21 days.

#### 2.4.3. Abiotic Degradation under Laboratory Conditions

For the abiotic degradation experiments, samples were first dried under vacuum at room temperature to a constant mass and then incubated in screw-capped vials with air-tight PTFE/silicone septa, containing 25 mL distilled water. The degradation experiment was conducted at 70 °C (±0.5 °C) as described elsewhere [29]. The temperature was selected according to the accelerated degradation test conditions of ISO 15814:1999 [30]. After a predetermined degradation time, the samples were separated from the degradation medium and dried under vacuum at room temperature.

### 2.5. Characterization of the Samples Before and After the Degradation Tests

#### 2.5.1. Visual Examination

The surface of the materials was examined using a Zeiss optical microscope (Opton Axioplan) equipped with a Nikon Coolpix 4500 color digital camera. Microscopic observations were performed at a magnification of 120x. Microscopic changes were analyzed by the scanning electron microscopy (SEM) method. SEM studies were performed using the Quanta 250 FEG (FEI Company, Fremont, CA, USA) high-resolution environmental scanning electron microscope operated at 10 kV acceleration voltage. The samples were observed without coating under low vacuum (80 Pa) by using a secondary electron detector (large field detector).

#### 2.5.2. Gel Permeation Chromatography (GPC) Analysis

The molar mass and molar mass dispersity of the samples studied were determined by the GPC method, which was performed with a chloroform solution at 35 °C and a flow rate of 1 mL/min using the Spectra Physics 8800 solvent delivery system with two Mixed C Styragel columns with a mixed bed, a linear range of *M_w_* 200–2,000,000, and the Shodex SE 61 refractive index detector. Sample solutions in chloroform (10 µL, 3% w/v) were injected into the system. Polystyrene standards (Calibration Kit S-M-10, Polymer Laboratories) with a narrow molar mass dispersity were used to generate the calibration curve.

#### 2.5.3. Thermal Properties

Thermal characteristics of the received materials were determined using the TA DSC 2010 apparatus (TA Instruments, New Castle, DE, USA). The first calorimetric trace (I-scan, first heating run) in which the thermal history was suppressed and the third calorimetric trace (III-scan, second heating run) were acquired from –80 °C to 200 °C at a heating rate of 20 °C/min. All the experiments were performed under a nitrogen atmosphere, with a constant nitrogen flow rate of 50 mL/min. The instrument was calibrated with indium standards. The melting temperature (*T_m_*) was considered as the temperature of the melting endotherm maximum and the glass transition temperature (*T_g_*) as the midpoint of the specific heat step associated with the transition. Thermogravimetric analysis (TGA) was performed with the TGA/DSC1 Mettler-Toledo thermal analyzer at a heating rate of 10 °C/min in a stream of nitrogen (60 mL/min). The obtained TGA data were analyzed with the Mettler-Toledo Star System SW 9.30.

#### 2.5.4. Nuclear Magnetic Resonance (NMR) Measurements

^1^H NMR spectra were recorded using a Bruker-Advance spectrometer (Fremont, CA, USA). Each spectrum was collected with 64 scans, 11 μs pulse width, and 2.65 s acquisition time. The spectrometer operated at 600 MHz using tetramethylsilane (TMS) as the internal standard and CDCl_3_ as the solvent.

## 3. Results and Discussion

The addition of a natural filler to the biodegradable polymer matrix increases the application possibilities of ecoproducts, such as packages, disposables tableware, acoustic, and heat insulation, as well as in the transport industry, such as interior door panels, dashboard elements, etc. obtained from those materials. An economical product is of considerable importance for manufacturers, but the ability to change the properties of the product by using different amounts of filler can create new application perspectives. The presence of the filler and its amount can influence the material degradation rate [31]. Three different composting systems used in this case study provide the complete information about material behavior during organic recycling. In the laboratory condition, the organic recycling of the investigated materials was assessed in the mature compost using the Micro-Oxymax respirometer. The water present in all composting systems initiates the hydrolysis process in the investigated materials. Study of the hydrolytic degradation conducted in the laboratory conditions facilitates an understanding of the degradation processes that occur in composting conditions [32].

### 3.1. Cork Effect on the Degradation Process

**Figure 1:**
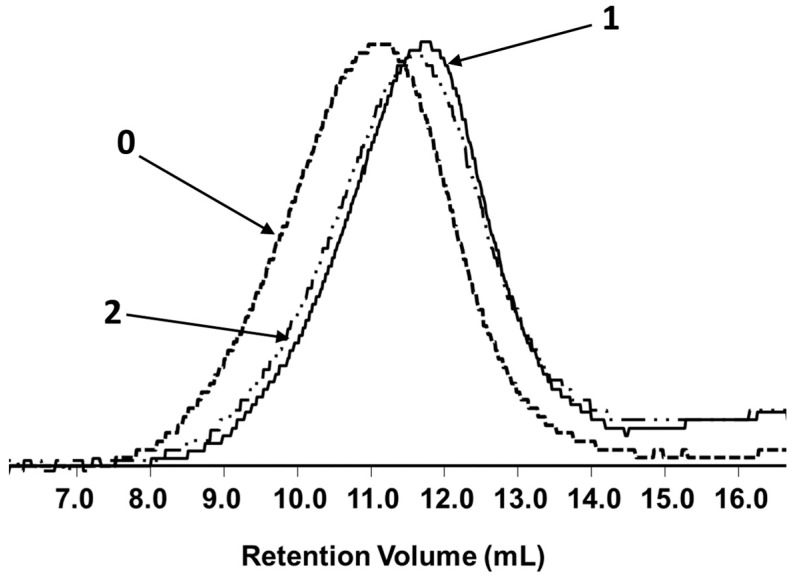
Overlay of selected GPC elugrams of pure P(3HB-*co*-4HB) before (0) and after processing (1) as well as the P(3HB-*co*-4HB) matrix with 30% cork (2).

**Figure 2:**
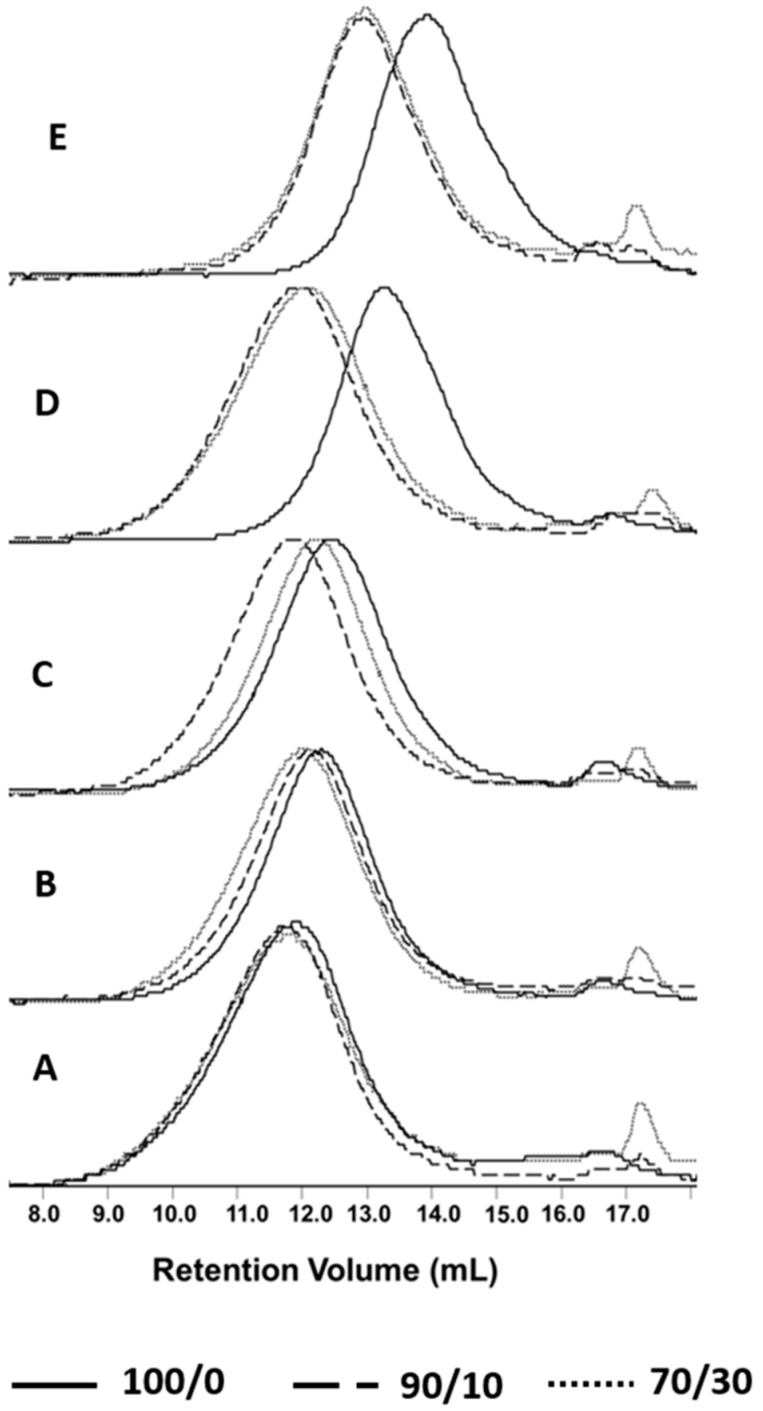
Overlay of selected GPC elugrams of the pure P(3HB-*co*-4HB) (100/0) and P(3HB-*co*-4HB)/cork composites with the mass ratio of 90/10 and 70/30, respectively, before (**A**) and after 21 days of degradation in a respirometer (**B**), BIODEGMA (**C**), container (**D**) and water (**E**).

The molar mass changes provide information about differences in the degradation progress of investigated materials after incubation in the abovementioned environments. The introduction of the natural filler into the polymer matrix slightly influenced the samples during processing (Figure 1) and significantly affected their degradation, because it reduced the molar mass loss during degradation (Figure 2, Table 2).

After 21 days, independently from the degradation environments, the neat P(3HB-*co*-4HB) matrix showed a more noticeable decrease in molar mass as compared to composites. Furthermore, the results of GPC analysis showed the order of shifts in the GPC traces to higher retention volume values for the molar mass of the pure P(3HB-*co*-4HB) matrix degraded in the water > container system > BIODEGMA system > respirometer. Because of the specificity of the environments, humidity played an important role during the degradation of the investigated materials. The KNEER system with closed water circulation and the biomass aeration system provide more favorable conditions for the growth of thermophilic organisms. In this system, enzymatic degradation may also play an important role due to the higher humidity of the compost [33]. After incubation in water and in the container system, where moisture was the highest, the shifting of the GPC traces of the pure P(3HB-*co*-4HB) matrix to higher retention volume values was clearly observed.

The degradation of composites was much slower. The composite samples showed a decrease in molar mass only after 21 days of degradation in water at 70 °C. This phenomenon can be explained by the properties of the filler. The characteristics of the cork result from its cellular structure, primarily its cell dimensions and topology, and from the chemical composition of the cell wall, which is composed of 53% suberin and 26% lignin. Lopes et al. showed that the molar mass (*M_n_*) of the suberin extracts determined by vapor pressure osmometry ranged from 528 to 968 g/mol [34]. This explains the presence of the small peak on the elution curves in the range of a low molar mass (high retention volume), which was especially apparent for P(3HB-*co*-4HB)/cork (70/30) samples. Suberin is a hydrophobic, elastic, and fire-retardant substance, and because of its impermeability and buoyant ability, it can inhibit the degradation of composites containing cork [35]. Cork containing suberin protects against the migration of water, microbial attack, and exposure to heat [36]. These properties may slow down not only the hydrolytic degradation process, but also the overall degradation of the compost, where microorganisms play an important role in addition to water. Interestingly, there was only a small difference in the molar mass reduction of the composite matrix and the pure P(3HB-*co*-4HB) matrix regarding the degradation in the respirometer; however, tendency was maintained. These differences could be caused by the use of mature compost in the respirometric tests. Further, after incubation in the BIODEGMA system, a difference in the degree of degradation was noted for both composites. The decrease in molar mass for the P(3HB-*co*-4HB)/cork composite with a mass ratio of 70/30 was more apparent and comparable to the degradation in the respirometer. This phenomenon observed after degradation in the BIODEGMA system could be explained by the heterogeneity of composted waste fractions and the influence of the presence of mixed municipal waste in the composted material as mentioned before.

### 3.2. Material Examination and Failure Analysis

**Figure 3:**
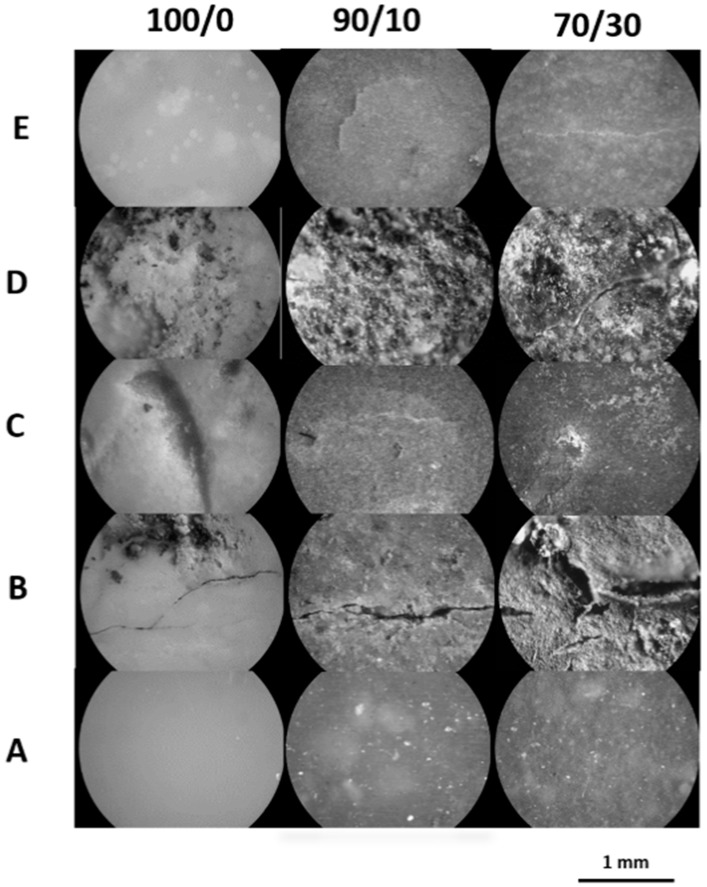
Photomicrographs (120x) of the pure P(3HB-*co*-4HB) (100/0) and P(3HB-*co*-4HB)/cork composites with a mass ratio of 90/10 and 70/30, respectively, before (**A**) and after 21 days of degradation in a respirometer (**B**), BIODEGMA (**C**), container (**D**) and water (**E**).

Microscopic evaluation of the investigated samples after 21 days of degradation in the determined environments indicated small differences in the material surface between the pure matrix and composites (Figure 3).

Microscopy images show that for pure P(3HB-*co*-4HB), less damage was found on the surface, which together with a higher molar mass loss (see Figure 2) may indicate a greater contribution to hydrolytic degradation. The abiotic degradation experiment in water at 70 °C led to erosion, as evidenced by the local discoloration (most visible for the pure P(3HB-*co*-4HB) matrix) due to possible crystallization of degradation products formed during hydrolysis inside the samples (see Table 3) [37]. Small cracks were also observed for composites because of water absorption during hydrolytic degradation.

**Figure 4:**
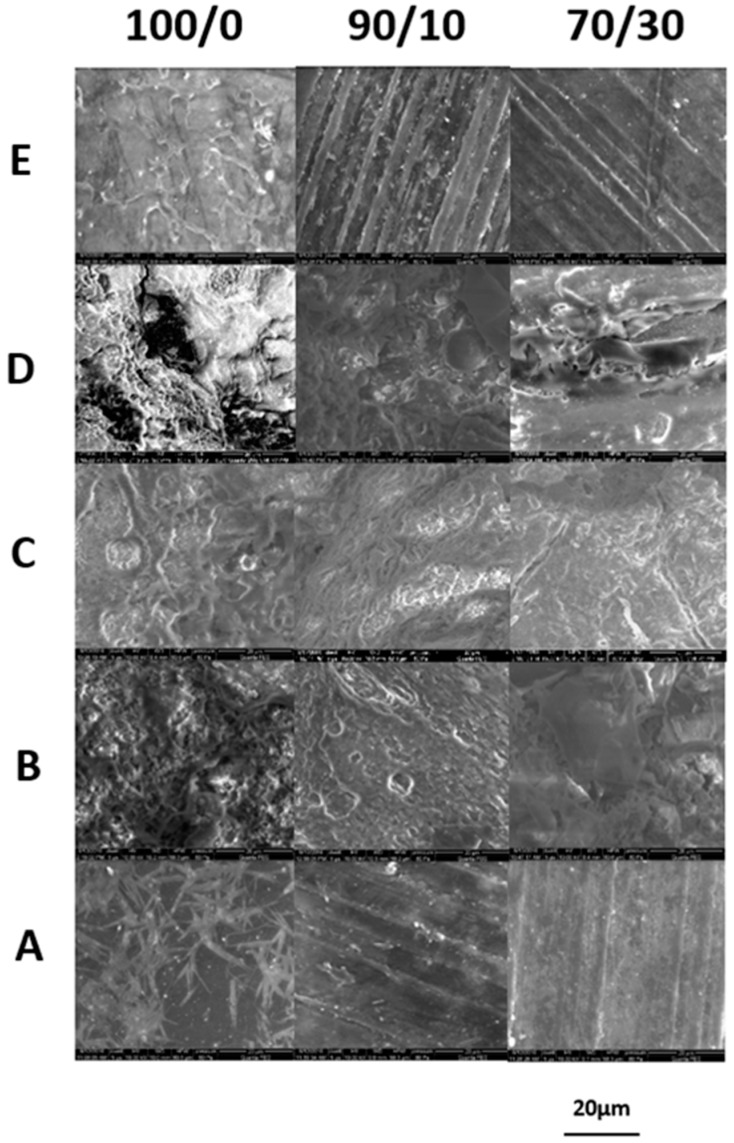
SEM micrographs (5000x) of the pure P(3HB-*co*-4HB) (100/0) and P(3HB-*co*-4HB)/cork composites with a mass ratio of 90/10 and 70/30, respectively, before (**A**) and after 21 days of degradation in a respirometer (**B**), BIODEGMA (**C**), container (**D**) and water (**E**).

The SEM analysis (Figure 4) showed that the degradation media excluding water penetrated the surface of the samples, especially in the case of composites; this indicates the dominance of enzymatic degradation. This is also evidenced by the small loss of molar mass (see Figure 2), which is typical during the initial stage of biodegradation. In SEM images on the micrometer scale, during degradation in all studied environments, microcracks were observed on the entire surfaces of all the examined composites. The most radical changes in the sample surface together with only slight changes in molar mass (see Figure 2 and Figure 4) were observed after 21 days of incubation in the Micro-Oxymax respirometer and in the container system, where the highest impact of enzymatic degradation was observed. In these samples, many pinholes, cavities, and cracks were visible. In the BIODEGMA system, these changes were less visible on the sample surface together with some changes in the molar mass (see Figure 2 and Figure 4), especially for the P(3HB-*co*-4HB)/cork composite with a mass ratio of 70/30. This is because the BIODEGMA system belongs to the type of composting systems in which the input material is undefined. This can lead to differences in the humidity of the composted material, and as a result, the process temperature changes in this system.

### 3.3. Thermal Behavior of the Investigated Samples during Degradation

The thermal decomposition curves of the pure P(3HB-*co*-4HB) matrix before and after degradation in the investigated environments followed a single mass loss step. For the composites, the second step of the mass loss occurred in the range related to the thermal degradation of cork, which reached the maximum decomposition temperature (*T_max_*) at 419 °C. Furthermore, the addition of the natural filler increased the thermal stability of the obtained materials (Table 3). After degradation, this same phenomenon was observed for all investigated samples and for the pure P(3HB-*co*-4HB) matrix.

During 21 days of incubation in all investigated environments, the formation of P(3HB-*co*-4HB) degradation products can be assumed. Abiotic hydrolysis is mostly considered to be the main degradation step as high humidity and temperatures enable the cleavage of ester linkages by water uptake, thus causing reduction in the molar mass. The oligomers and acids formed during degradation of the investigated samples were present in all environments depending on the degradation degree [38]. Some of the degradation products could have migrated into the degradation medium, while the other products remained in the material [39,40]. The presence of the acidic degradation products and the compounds derived from the filler in the samples was responsible for increasing the thermal stability. The cork extract contains natural thermal oxidation stabilizers, such as suberin, which acts not only as a plasticizer, but also has antioxidant properties due to its high content of phenolic compounds, which are known to be capable of scavenging radicals. These molecules could be responsible for the stabilizing effect observed for cork composites [41]. After 21 days of incubation in water at 70 °C and in the KNEER system, where the humidity was the highest, the largest increase in thermal stability was observed for the pure matrix sample. These results overlap with the small decrease in the molar mass in the same environments, which would lower the thermal stability of the material. Hence, we can conclude that the respective presence of the degradation products inside the sample influenced the thermal stability of the material.

During degradation of all the investigated materials with different cork mass ratios, significant changes occurred in the thermal behavior.

The thermal properties of the investigated samples by DSC for the first heating run are listed in Table 4.

The *T_g_* and the melting enthalpy (*ΔH_m_*) of the composites were higher than those for the pure matrix. When the cork content increased, the *T_g_* of P(3HB-*co*-4HB) increased, indicating the occurrence of physical entanglement. The cork plays a role of an antiplasticizer, which increased the glass transition temperature of the investigated materials. It is apparent from the DSC traces of the pure samples that for the composites with 10% and 30% cork content, the melting peaks were broader, and a double endotherm peak was observed in both composites (data not shown). This phenomenon may be caused by the polymorphism tendency of the composites toward filler nucleation. This tendency was more apparent for the samples with a higher amount of cork. The obtained results indicated that the presence of cork as the nucleation agent initiates crystallization by nucleation. Multiple melting peaks in the DSC curves observed for the investigated materials have also been reported for many semicrystalline polymers. This can be explained by the formation of a dual population of crystallites during processing [38]. After degradation of the pure P(3HB-*co*-4HB) matrix, especially in an environment with high humidity, a significant increase in *ΔH_m_* was observed. This finding suggests that the crystallization of the material can be explained as a consequence of the degradation mechanism. The different forms of degradation products can also play roles as nucleation agents.

The melting enthalpy of the composites after degradation showed a downward trend. According to Rydz et al., amorphous forms of the polymer absorb water quicker than crystalline forms [42]. Hence, for the composites with higher *ΔH_m_* before degradation, the degradation process slowed down. This was confirmed by the changes in the molar mass (see Figure 2).

### 3.4. Structural Characterisation of P(3HB-co-4HB)

**Figure 5:**
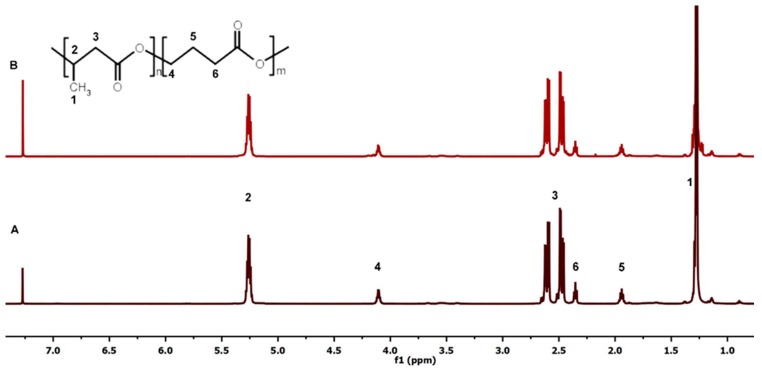
^1^H NMR spectrum of P(3HB-*co*-4HB) before (**A**) and after 21 days of degradation in distilled water (**B**).

The composition of P(3HB-*co*-4HB) was determined based on ^1^H NMR analysis. The ^1^H NMR spectrum of P(3HB-*co*-4HB) before degradation showed signals corresponding to the protons of 3-hydroxybutyrate constitutional repeating units (3HB, signals 1-3) and signals corresponding to the protons of 4-hydroxybutyrate constitutional repeating units (4HB, signals 4–6), two components of the copolymer (Figure 5). The spectrum also showed low signals corresponding to the dicarboxylic acid—azelaic acid. Azelaic acid derivatives are usually added to PHAs as a plasticizer [43,44]. Furthermore, small amounts of crotonic acid and its oligomers, products of P3HB degradation, and poly(3-hydroxyhexanoate), an additional product of microbial synthesis, were identified. Additives and impurities did not exceed 2% of the total polymer and did not significantly affect the course of (bio)degradation.

The analysis by ^1^H NMR (see Figure 5, based on the methylene moiety of the P4HB and methyl group of the P3HB) after 21 days of degradation indicated only slight changes (no more than 1%) in the compositions of the copolymer investigated in the biodegradation experiment.

## 4. Conclusions

The obtained results indicate that the addition of cork as a natural filler to P(3HB-*co*-4HB) changed the properties of the composites in comparison to those of the pure matrix. This particularly applied to the thermal behavior of the samples. Thermal stability of the composites was higher than that for the pure matrix. Furthermore, this value increased after 21 days of incubation in all environments. The presence of cork also significantly affected the degradation profile of the obtained composites. The degradation rate of the pure matrix was higher than that of the composites in all investigated environments. In the case of pure P(3HB-*co*-4HB), higher molar mass loss and less damage were found on the surface. This finding indicates a greater contribution to hydrolytic degradation. The cork properties inhibit the penetration of water into the polymer matrix in composites, because this filler is impermeable to liquids [21]. Changes on the surface of composites together with a small loss of molar mass indicate that because the occurrence of hydrolysis was difficult, enzymatic degradation and/or mechanical changes due to the presence of filler could have prevailed in the composting conditions. The obtained results suggest that the addition of cork as a natural filler in various mass ratios to the composites generates the possibility of obtaining products with different life expectancies.

## Figures and Tables

**Table 1:** Processing parameters for sample preparation.

P(3HB-*co*-4HB)/Cork (Mass Ratio)	Temperature of Plasticizing Zone (°C)	Injection Temperature (°C)	Injection Pressure (bar)
100/0	140	140	350
90/10	140	140	450
70/30	140	140	650

**Table 2:** Molar mass before and after 21 days of degradation of pure P(3HB-*co*-4HB) and P(3HB-*co*-4HB)/cork composites.

P(3HB-*co*-4HB)/Cork (Mass Ratio)	Environment	*M_w_* [g/mol]	*M_w_/M_n_*
100/0	Before degradation	501,000	5.0
90/10		554,000	5.0
70/30		560,000	5.1
100/0	Respirometer	189,000	3.7
90/10		230,000	3.6
70/30		330,000	3.9
100/0	BIODEGMA	143,000	4.0
90/10		341,000	4.1
70/30		218,000	4.0
100/0	Container	49,000	3.2
90/10		479,000	4.2
70/30		455,000	5.4
100/0	Water	19,000	3.3
90/10		62,000	3.1
70/30		68,000	3.4

**Table 3:** Thermogravimetric parameters before and after 21 days of degradation of pure P(3HB-*co*-4HB) and P(3HB-*co*-4HB)/cork composites.

P(3HB-*co*-4HB)/Cork (Mass Ratio)	Environment	*T_max_* (°C)	*T*_5%_ (°C)
100/0	Before degradation	243	217
90/10		251/409	227
70/30		255/412	237
100/0	Water	297	275
90/10		289/413	277
70/30		295/422	277
100/0	Container	271	249
90/10		266/414	245
70/30		260/413	240
100/0	BIODEGMA	258	231
90/10		266/411	251
70/30		272/419	256
100/0	Respirometer	263	240
90/10		287/413	251
70/30		280/420	261

*T_max_*—maximum decomposition temperature, *T*_5%_—the temperature corresponding to 5% mass loss.

**Table 4:** Calorimetric parameters of P(3HB-*co*-4HB) and P(3HB-*co*-4HB)/cork composites before and after 21 days of degradation in different environments.

P(3HB-*co*-4HB)/Cork (Mass Ratio)	Environment	*T_g_* (°C)	*T_m_* (°C)	*ΔH* (J/g)
100/0	Before degradation	–9.4	140.5/179.5	46.0/0.9
90/10		–0.6	128.9/160.9	74.5
70/30		5.4	132.1/152.0/160.5	77.8
100/0	Water	–9.0	137.1/140.6/148.4	71.3
90/10		–2.5	141.9/157.8	66.9
70/30		1.8	134.9/153.8	68.1
100/0	Container	–4.5	142.1/154.3	70.2
90/10		1.2	130.6/151.9	73.0
70/30		5.4	135.8/153.3	70.4
100/0	BIODEGMA	–4.3	147.0	50.4
90/10		–1.2	130.5/147.6	57.3
70/30		5.3	131.6/156.4	67.8
100/0	Respirometer	–3.3	140.3/160.6	83.3
90/10		–0.8	129.1/148.5	56.1
70/30		3.5	132.6/149.2	72.0

*T_m_*—melting temperature, *ΔH_m_*—melting enthalpy (first heating scan, 20 °C/min), *T_g_* (second heating scan after rapid cooling, 20 °C/min). (Thermograms in the Supplementary Materials Figure S1).

## Supplementary Materials

The following are available online at https://www.mdpi.com/2073-4360/11/3/547/s1, Figure S1: DSC plot of P(3HB-co-4HB)/cork composites (100/0), (90/10), (70/30) before and after 21 days of degradation in different environments.
