# Supplementary material for: (Bio)degradable Polymeric Materials for Sustainable Future—Part 2: Degradation Studies of P(3HB-co-4HB)/Cork Composites in Different Environments
Source: Polymers (Basel). 2019 Mar 22;11(3):547. doi: 10.3390/polym11030547 (PMC6473375; doi:10.3390/polym11030547)

DSC plot of P(3HB-co-4HB)/cork composites (100/0), (90/10), (70/30) before degradation.

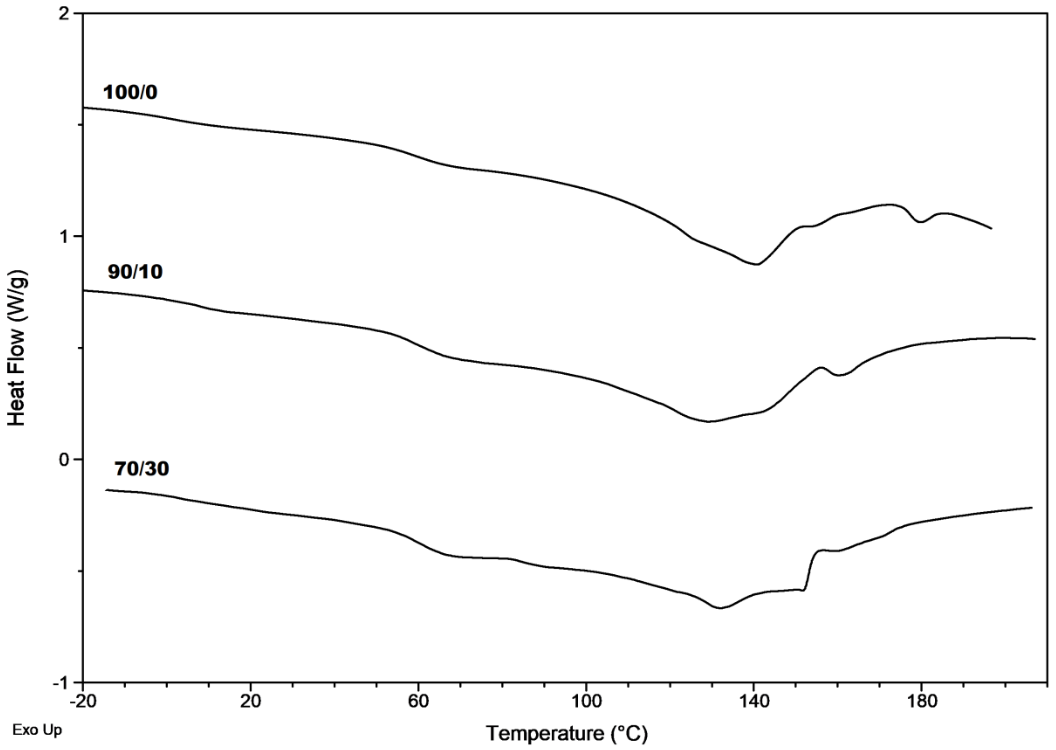

DSC plot of P(3HB-co-4HB)/cork composites (100/0), (90/10), (70/30) after 21 days of degradation in the water.

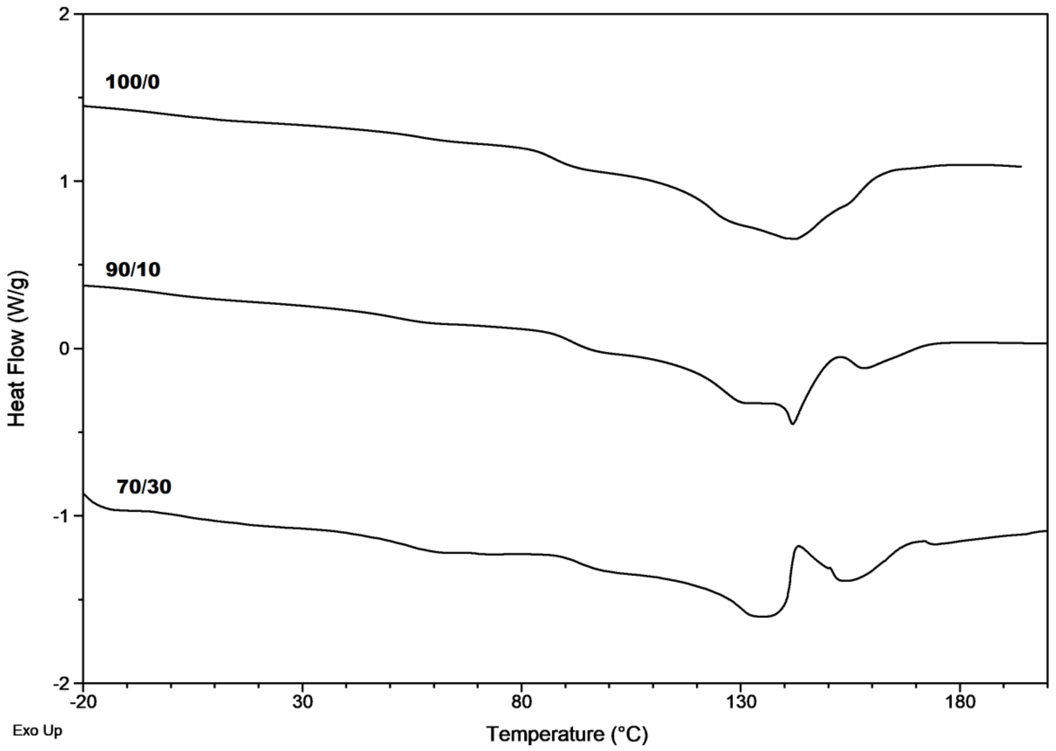

DSC plot of P(3HB-co-4HB)/cork composites (100/0), (90/10), (70/30) after 21 days of degradation in the container.

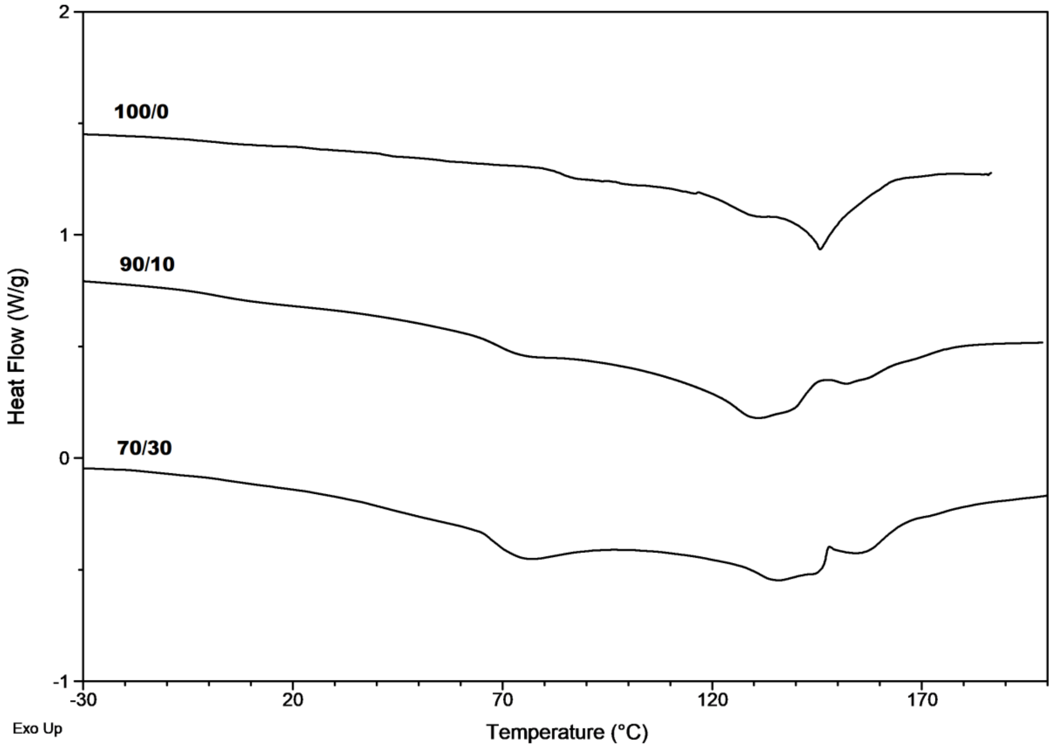

DSC plot of P(3HB-co-4HB)/cork composites (100/0), (90/10), (70/30) after 21 days of degradation in the BIODEGMA.

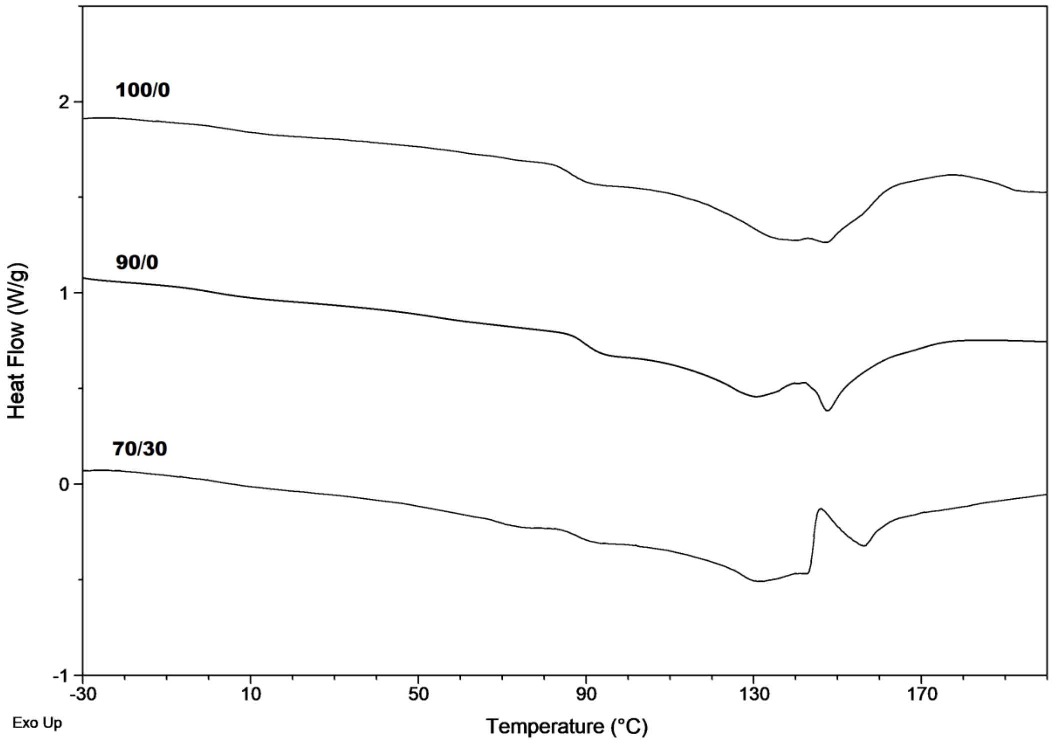

DSC plot of P(3HB-co-4HB)/cork composites (100/0), (90/10), (70/30) after 21 days of degradation in the respirometer.

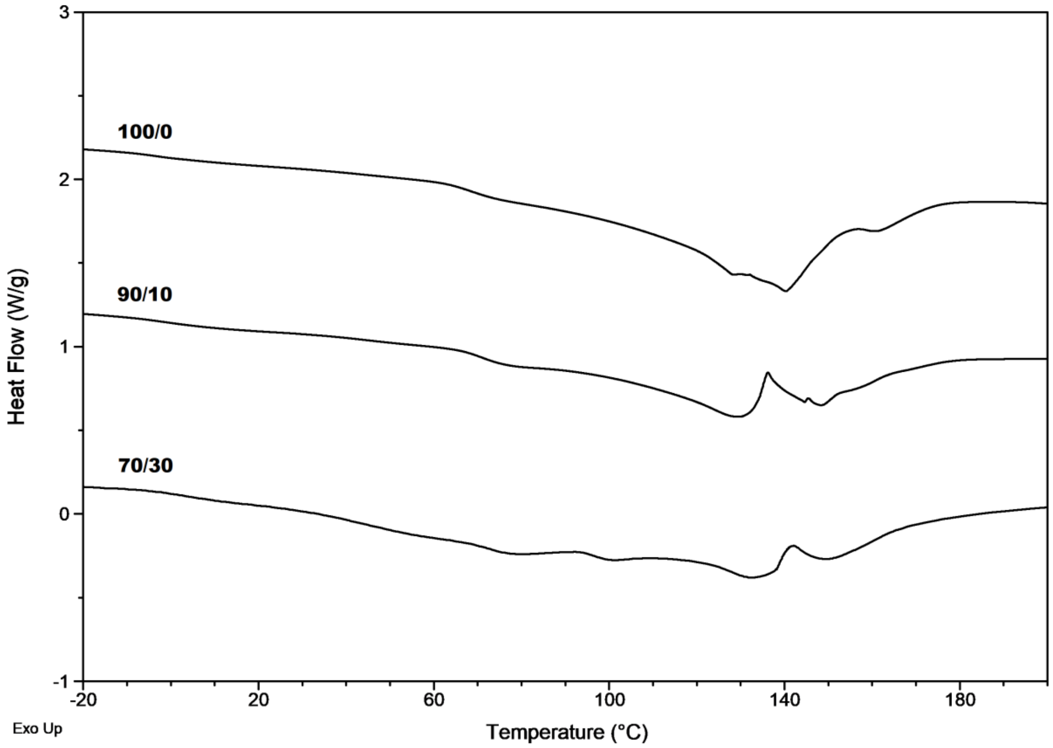

Supplement: Supplementary file 1 [file polymers-11-00547-s001.pdf]
